# Social factors affecting seasonal variation in bovine trypanosomiasis on the Jos Plateau, Nigeria

**DOI:** 10.1186/1756-3305-6-293

**Published:** 2013-10-10

**Authors:** Ayodele O Majekodunmi, Akinyemi Fajinmi, Charles Dongkum, Kim Picozzi, Ewan MacLeod, Michael V Thrusfield, Alexandra P M Shaw, Susan C Welburn

**Affiliations:** 1 Division of Pathway Medicine and Centre for Infectious Diseases, School of Biomedical Sciences, College of Medicine and Veterinary Medicine, The University of Edinburgh, Chancellor’s Building, 49 Little France Crescent, Edinburgh EH16 4SB, UK; 2 Nigerian Institute for Trypanosomiasis Research, P.M.B. 1303, Vom, Plateau State, Nigeria; 3 Veterinary Clinical Sciences, Royal (Dick) School of Veterinary Studies, College of Medicine and Veterinary Medicine, Easter Bush Veterinary Centre, Roslin, Midlothian EH25 9RG, UK

**Keywords:** African animal trypanosomiasis, Emerging disease, Risk factors, Seasonal dynamics, Nigeria, PCR, Tsetse, Jos plateau, Fulani, Pastoralist, Transhumance

## Abstract

**Background:**

African Animal Trypanosomiasis (AAT) is a widespread disease of livestock in Nigeria and presents a major constraint to rural economic development. The Jos Plateau was considered free from tsetse flies and the trypanosomes they transmit due to its high altitude and this trypanosomiasis free status attracted large numbers of cattle-keeping pastoralists to the area. The Jos Plateau now plays a major role in the national cattle industry in Nigeria, accommodating approximately 7% of the national herd, supporting 300,000 pastoralists and over one million cattle. During the past two decades tsetse flies have invaded the Jos Plateau and animal trypanosomiasis has become a significant problem for livestock keepers. Here we investigate the epidemiology of trypanosomiasis as a re-emerging disease on the Plateau, examining the social factors that influence prevalence and seasonal variation of bovine trypanosomiasis.

**Methods:**

In 2008 a longitudinal two-stage cluster survey was undertaken on the Jos Plateau. Cattle were sampled in the dry, early wet and late wet seasons. Parasite identification was undertaken using species-specific polymerase chain reactions to determine the prevalence and distribution of bovine trypanosomiasis. Participatory rural appraisal was also conducted to determine knowledge, attitudes and practices concerning animal husbandry and disease control.

**Results:**

Significant seasonal variation between the dry season and late wet season was recorded across the Jos Plateau, consistent with expected variation in tsetse populations. However, marked seasonal variations were also observed at village level to create 3 distinct groups: Group 1 in which 50% of villages followed the general pattern of low prevalence in the dry season and high prevalence in the wet season; Group 2 in which 16.7% of villages showed no seasonal variation and Group 3 in which 33.3% of villages showed greater disease prevalence in the dry season than in the wet season.

**Conclusions:**

There was high seasonal variation at the village level determined by management as well as climatic factors. The growing influence of management factors on the epidemiology of trypanosomiasis highlights the impact of recent changes in land use and natural resource competition on animal husbandry decisions in the extensive pastoral production system.

## Background

The Jos plateau in North-Central Nigeria covers an area of 8000 km^2^ at an average altitude of 1,280 m. The Jos Plateau has previously been considered free of AAT, the altitude being assumed to be too high to permit tsetse colonisation and rendering the Plateau free of the tsetse vector and the trypanosomes they transmit [1,2]. However, the Plateau is surrounded on all sides by tsetse-infested lowlands that include Human African Trypanosomiasis (HAT) or sleeping sickness foci and that are endemic for animal trypanosomiasis. The absence of disease and abundant pasture on the Jos Plateau attracted many pastoral cattle herders and the plateau is now an area of intense animal production playing a significant role in the national cattle industry. However, during the last two decades trypanosomiasis has been reported on the Plateau and there has been a consequent reduction in both cattle numbers and economic potential for cattle grazing on the plateau. From closer examination of reports from isolated village surveys and from surveys undertaken in Local Government Areas, it is clear that both tsetse flies and AAT have been present on the Jos Plateau at least since the 1980s and that the tsetse free status of the Plateau is no longer valid [3]. We have recently reported on the prevalence of bovine trypanosomiasis in this area [4] and the present work focuses on seasonal variation and the social factors influencing the epidemiology of this disease.

## Methods

### Study design

**Figure 1:**
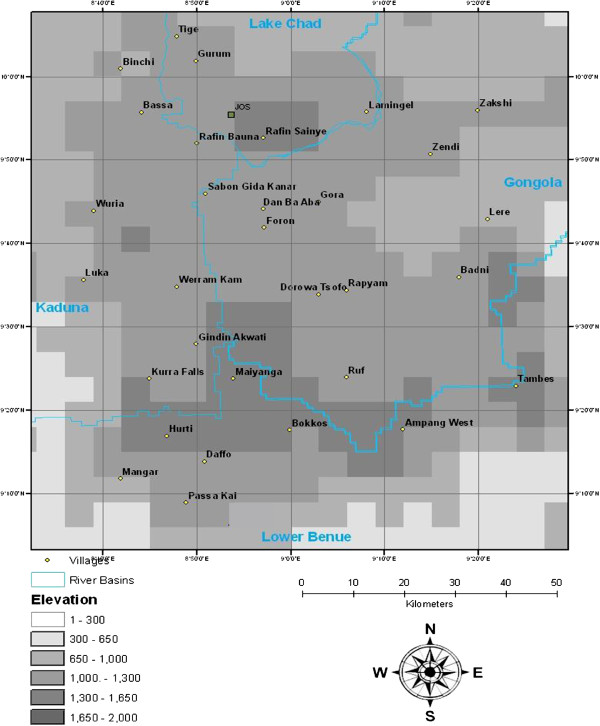
Study area showing selected villages.

A longitudinal two-stage stratified cluster sampling design was applied in which individual cattle represented the ultimate sampling unit and individual villages were clusters. In the first stage, villages were stratified by their river basin and selected using probability proportional to size. For the second stage, a fixed number of cattle were selected in each village that was to be included in the study. An assumed mean trypanosomiasis prevalence of 24% [5-8] and rate of homogeneity of 0.115 [9] were applied and entered into CSurvey (©UCLA, 2007) to determine the minimum number of clusters required and to test the final sample size. A minimum number of thirty individual clusters (villages), with eighty cattle sampled per cluster were required to estimate AAT disease prevalence with a confidence level of 95%. Thirty villages were selected by applying a 15 km × 20 km grid across the study area, with one village per square identified as indicated in Figure 1.

Following assessment of the available scientific literature concerning seasonal variations in tsetse and trypanosomiasis migration patterns on the Jos Plateau in particular and more generally in northern Nigeria [10] a three-point longitudinal survey was designed to examine seasonal variations in AAT, whereby sampling for AAT was undertaken during the dry season (March), early wet season (June) and the late wet season (October). Molecular diagnosis of trypanosome infections was undertaken using species-specific polymerase chain reactions (PCR) for *T. brucei brucei*, *T. congolense* savannah and *T. vivax* as previously described [4].

### Qualitative methods

Structured questionnaires are a standard method for collecting survey data but large-scale surveys and long questionnaires can be biased, unreliable and difficult to administer and analyse. Participatory rural appraisal (PRA) uses a range of qualitative sample survey methods to gather information by allowing local people to share and analyse their knowledge and experiences and to make plans based on this information [11]. It is quicker and more cost effective than long-term studies and uses and assimilates a wide range of multidisciplinary information, enabling on the spot assessment and direct response to actual village level problems. It also facilitates collection of standardised field data for use in more sophisticated databases. These qualitative methods may be used on their own or alongside more formal quantitative methodologies. Structured questionnaires incorporating several PRA techniques were administered to livestock owners in individual interviews and focus group discussions focusing on animal husbandry, pastoral livelihoods and the factors affecting them. Qualitative and quantitative data on knowledge, attitude and practices of animal husbandry and disease control was gathered and used to analyse which social, economic, ecological and cultural factors influence animal health and disease control by herders.

Herders (Fulani and Indigenes) were unwilling to respond to questionnaires individually so the structured questionnaire outlined above was administered in focus group discussions with results for most sections recorded as village results. The focus groups consisted of all herders whose cattle had been sampled and group size varied from 5 – 25.

Exact binomial confidence intervals at 95% level of confidence [12] were calculated using CIA^©^ BMJ software. Chi squared (χ^2^) test (EpiInfo^TM^ CDC software) for independence was used to test the association between two categorical variables or nominal data where the basis of comparison is proportions [13].

### Ethical approval statement

The study was carried out with the full approval of cattle keepers, the Plateau State Ministry of Agriculture and the Nigerian Institute for Trypanosomiasis Research (NITR), a Federal Government body. The University of Edinburgh is a charitable body, registered in Scotland, with registration number SC005336.

## Results

**Figure 2:**
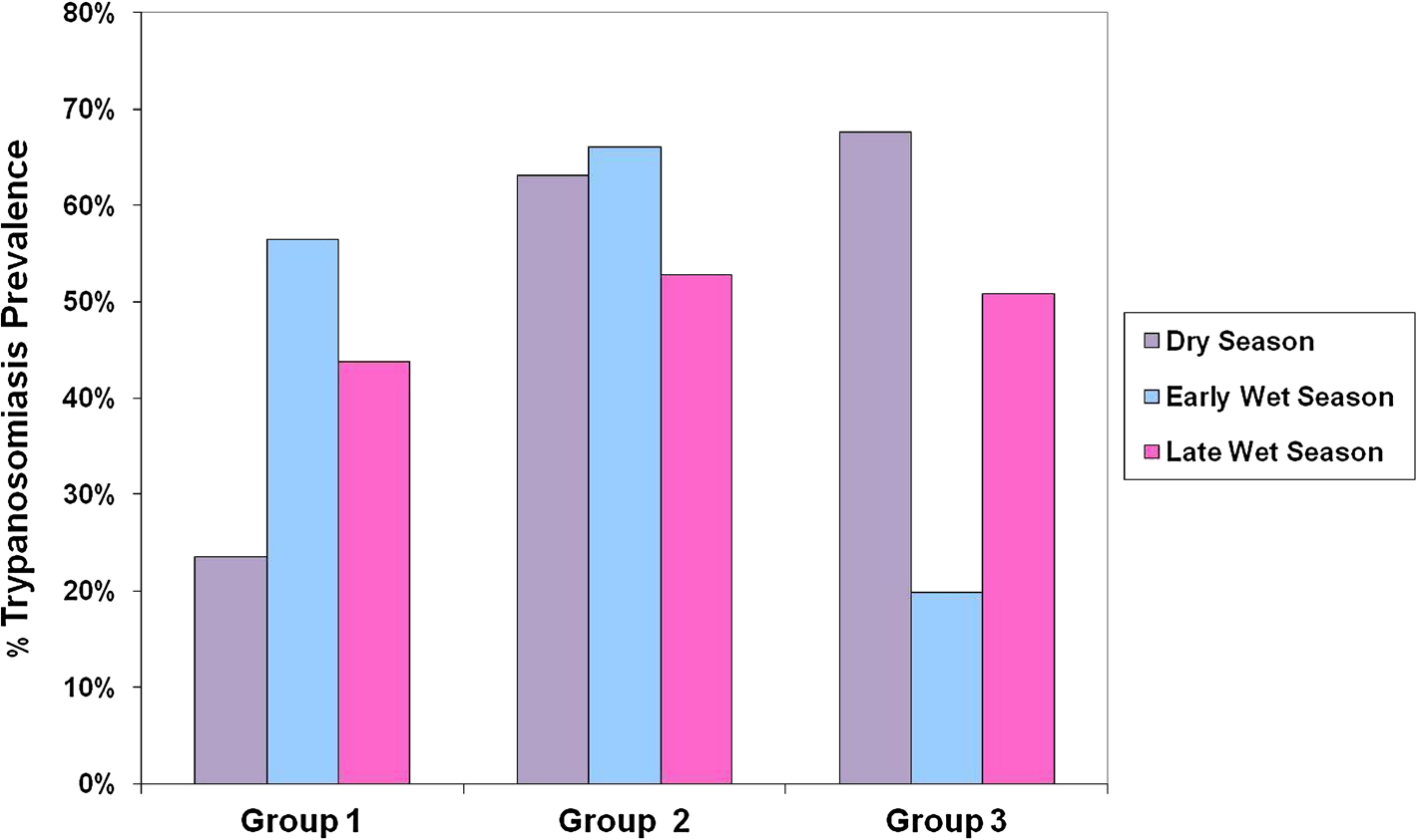
Three different seasonal variation patterns observed at village level.

**Figure 3:**
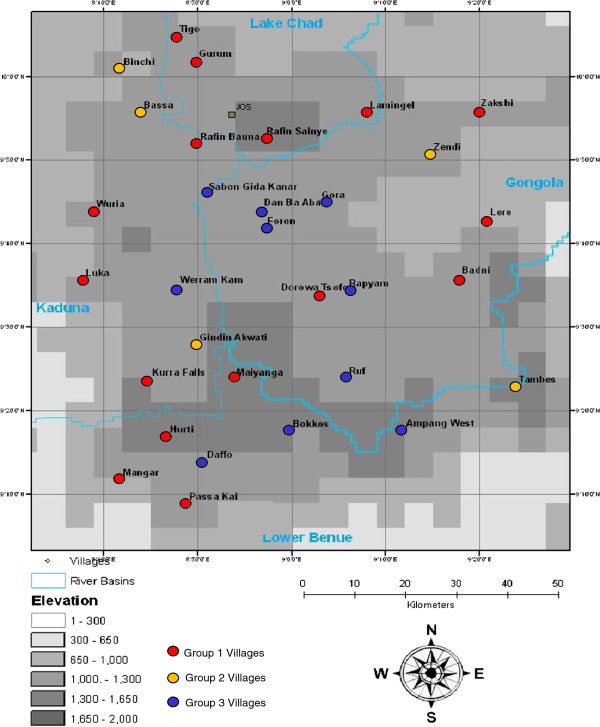
Distribution of villages with different seasonal variation patterns.

The overall prevalence of AAT across the Jos Plateau was very high (46.8%) and showed significant seasonal variation between the dry and late wet season (3% {0.16 – 5.9%} difference in proportions) and a wide range of AAT prevalence at village level (88% - 95.6%) as previously reported [4]. Three district patterns of seasonal variation were observed in village level data as shown in Table 1 and Figures 2 and 3.

**Table 1:** Prevalence of trypanosomiasis in seasonal variation groups with confidence intervals in brackets

	**African animal trypanosomiasis prevalence**			
** Group**	**Dry season**	**Early**	**Late**	**Annual**
		**Wet season**	**Wet season**	
Group 1	23.5%	56.4%	43.7%	41.6%
	(9.0% - 38.0%)	(36.5% - 76.3%)	(29.4% - 58.0%)	(28.7% - 54.5%)
Group 2	63.1%	66.0%	52.7%	62.7%
	(18.4% - 100%)	(36.2% - 95.8%)	(8.2% - 97.2%)	(30.6% - 94.8%)
Group 3	67.6%	19.9%	50.9%	46.1%
	(54.6% - 80.6%)	(8.0% - 31.8%)	(31.0% - 70.8%)	(36.5% - 55.7%)

### Group 1

Fifteen of the 30 study villages (50%) fell into this group. Cattle AAT showed significant seasonal variation in these villages where the prevalence of trypanosomiasis is lowest in the dry season and increases significantly in the wet season (χ^2^ = 211, p = <0.001).

### Group 2

Five of the 30 study villages (16.7%) fell in this group. Cattle AAT in these villages showed no significant seasonal variation where the prevalence of trypanosomiasis remains at a constant level all year round. (χ^2^ = 1.5, p = 0.173).

### Group 3

Ten of the 30 study villages (33.3%) fell into this group. Cattle AAT showed significant seasonal variation in these villages, the prevalence of trypanosomiasis being highest in the dry season, decreasing as the wet season begins and increasing again towards the end of the wet season. (χ^2^ = 518, p = <0.001).

### Transhumance

**Figure 4:**
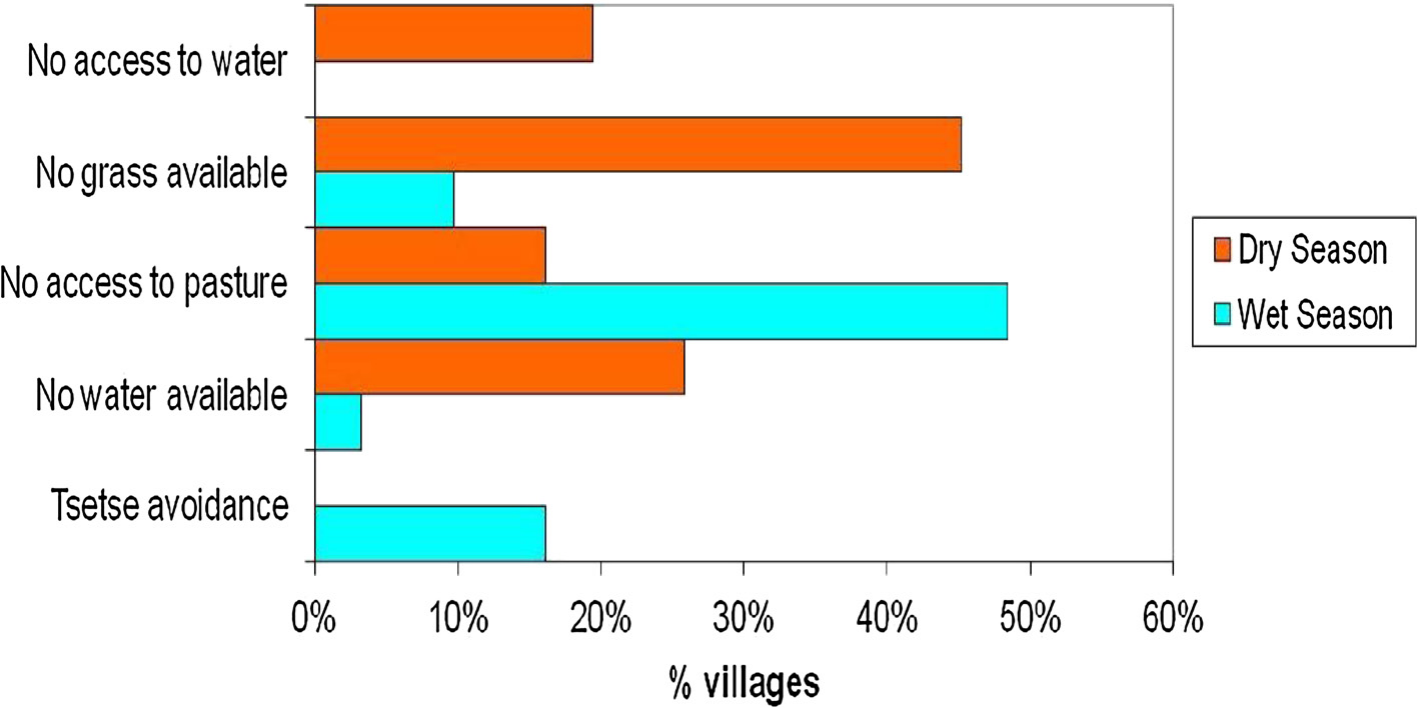
Reasons given by cattle owners for undertaking seasonal migration.

**Figure 5:**
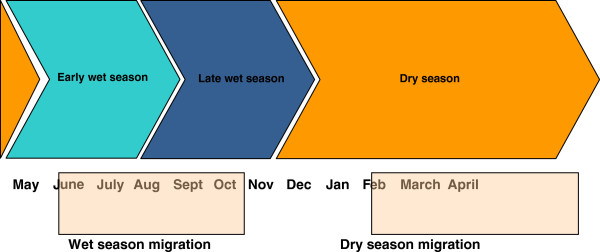
Seasonal calendar with migration periods.

**Figure 6:**
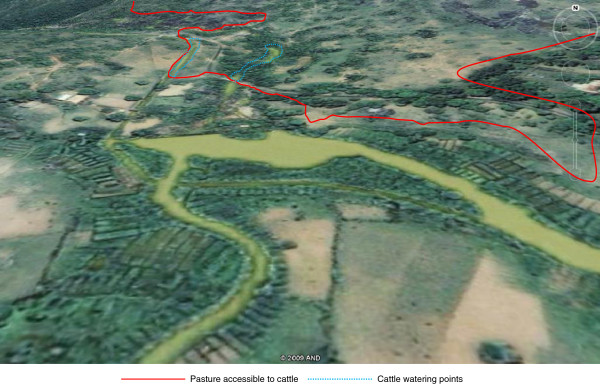
Wet season satellite image of Gashish River surrounded by vegetable plots (Google Earth, 2009).

Results from the qualitative surveys showed that of the 30 study villages, 22 villages (73.4%) practiced transhumance: 3 (10.0%) only during the dry season, 5 (16.7%) only during the wet season and 14 (46.7%) during both wet and dry seasons. Cattle keepers in eight villages did not practice transhumance. The reasons given by respondents for their migration practices in the dry and wet seasons are shown in Figure 4. Figure 5 illustrates the seasonal calendar and migration periods. Figure 6 shows the extent that pastoral access to pasture and water can be restricted by the presence of crops.

#### Dry season migration is practiced by herders in search of adequate pasture and water

Climatic conditions mean that there is very little grass or water available during the dry season. Pastoral access to these scant resources is further restricted by dry season farming activities practiced by the indigenous populations that are common on the Jos Plateau and take priority over cattle grazing. This means that even when grazing and/or water is available, cattle may not be able to access it and herders move them to areas with less land pressure.

#### Rainy season migration is practiced for

a) *Accessible grazing and water:* Pasture and water are widely available in the wet season, but uptake of more commercial and intensive farming methods and a higher proportion of cash crops mean that arable farmers increasingly demand more land and water for irrigation so herders are marginalised.

b) *Avoidance of farmers’ crops:* Increasing populations and land pressure have intensified farmer/pastoralist conflicts and increased the frequency of animals trampling crops. In many areas these issues have been resolved simply by asking pastoralists to remove their cows from the village from the time the crops appear above ground until harvest.

c) *Avoidance of tsetse and/or biting flies:* In areas with a severe fly nuisance, cattle are moved to more favourable areas of the Plateau or to nearby lowlands.

## Discussion

High variation in trypanosomiasis prevalence in cattle was observed at village level across the Jos Plateau. Seasonal variation was also pronounced at village level as determined by management as well as climatic factors.

The marked seasonal variations observed at village level led to classification of the villages into three distinct groups, as explained above. The seasons are as follows on the Plateau: Dry season (November - May); early wet season (May - August) and the late wet season (August - October). Two riverine tsetse species *Glossina tachinoides* and *G. palpalis palpalis* inhabit the Jos Plateau [3,14]. Expansion of the *G. p. palpalis* and *G. tachinoides* tsetse belts onto the Jos Plateau has been observed [15-17] and there is evidence of their persistence [3,8,18].

In Group 1 villages the prevalence of AAT in cattle was low in the dry season, peaked in the early wet season and decreased in the late wet season. This pattern follows the expected seasonal variation of tsetse transmitted trypanosomiasis in the sub-humid zone, whereby tsetse populations are low and their distribution is limited in the dry season and then as the wet season progresses tsetse populations increase and become more widely distributed [19].

In Group 2 villages, the prevalence of AAT remained consistent, year round with no significant differences observed in seasonal prevalence. This pattern is similar to that observed in areas where low populations of riverine tsetse flies restricted to riverine gallery forests were able to maintain year round transmission of trypanosomiasis [20-22], which indicates a persistent tsetse population transmitting AAT all year round, unaffected by seasonal variation. *The riverine tsetse* flies present on the Jos Plateau are highly adaptable to changing conditions as they are able to using micro-climatic niches [23-26] and opportunistic feeding behaviour [27], leading to increased density and expansion of this group of tsetse flies. In Group 2 villages, the persistence of gallery forests even during the dry season provides a suitable habitat throughout the year. These gallery forests often serve as human and cattle watering points, allowing close vector/host contact to be maintained between riverine tsetse and their hosts, providing a constant food supply and facilitating high transmission rates.

In Group 3 villages, AAT was observed to peak in the dry season, decrease dramatically in the early wet season and increase again in the late wet season. There are several drivers for this reversed epidemiological profile.

The long 7- month dry season on the Jos Plateau, is a period of intense stress for cattle with little food and water available; cattle are herded for between five and six kilometres per day in search of adequate grazing. Malnutrition and high stress increase both susceptibility to trypanosomiasis and promote negative outcomes of infection [28]. Nutrition is one of the key factors determining outcomes of trypanosomiasis infections since the degree of anaemia and growth retardation in infected cattle depends on protein and energy intake [29-31].

Secondly, agricultural expansion by farming communities has recently stimulated a rise in reclamation of pasture lands previously available for use by pastoralists. The introduction of irrigated farming systems has resulted in farmers developing vegetable plots around ponds and rivers that now restrict access of cattle to drinking water [32] and serve to exacerbate the impacts of the harsh dry season on nutrition and stress. In many villages, Fulani pastoralists have been forced to migrate to areas where there is lower land pressure and more abundant resources but which have significantly higher tsetse challenge to avoid community conflict and livestock mortality through stress and starvation [33,34]. Dialogue between pastoral and farming communities has resulted in the allocation of specific resources for use by cattle in some villages but this is considered to be to the advantage of the farmers and not pastoralists [35]. This study has observed, first hand, the restricted access to pasture for cattle in these villages (Figure 6). Extensive migration in response to reduced access to natural resources comes at a significant burden to pastoralists increasing susceptibility to trypanosomiasis due to the stress of trekking and exposes cattle to higher tsetse densities and greater challenge to novel strains of pathogenic trypanosomes.

The dry season on the Jos Plateau is associated with low humidity and high temperatures both of which have direct negative effects on tsetse population growth [36-38]. Tsetse correlations with trypanosomiasis prevalence are also dependent on dispersal and such harsh climatic conditions limit tsetse dispersal by forcing flies to congregate in gallery forests where temperatures are lower, humidity higher and there is abundant shade. In contrast, the favourable conditions in the wet season support increased tsetse populations and promote greater fly dispersal. Consequently, the dry season exhibits the highest relative tsetse abundance and the highest apparent density. This profile is most often seen in drier areas like the Guinea and Sudan savannah zones and was unexpected in the sub-humid zone of the Jos Plateau. The Jos Plateau is not well supplied with ground water and only a few streams and rivers persist throughout the dry season. Cattle and the small residual populations of tsetse flies concentrate at these points that provide high degrees of host-vector contact. Intensive cropping and erosion have destroyed much of the tsetse habitat in these areas of the Plateau, restricting available tsetse habitats to gallery forests. Kalu, 2001 [39] reports a similar situation in the neighbouring Benue State with high AAT prevalence of over 40% in the dry season and much lower AAT prevalence in the wet season (9 – 29%). Similar dry season peaks in AAT prevalence have been reported in Ethiopia attributed to habitat fragmentation [40].

The influence of management factors on the epidemiology of trypanosomiasis highlights a growing trend in Nigerian cattle production; the traditional low input extensive production system is under threat due to expansion of human and livestock populations and arable farming. Pasture and water are becoming increasingly scarce and pastoralists are responding with management practices that increase risk of AAT. The combination of harsh climatic conditions, natural resource conflict and increased tsetse challenge appear to promote very high transmission rates of trypanosomiasis on the Jos Plateau in the dry season. In the early wet season, food and water become more widely available and tsetse populations become more dispersed; improved condition in the animals and reduced vector-host contact leads to a reduction in trypanosomiasis prevalence. By the late wet season however, tsetse populations have increased considerably and despite increased dispersal, there is a higher tsetse challenge and consequent increase in trypanosomiasis prevalence in cattle.

## Conclusions

A longitudinal survey of bovine trypanosomiasis revealed a high overall prevalence of trypanosomiasis (46.8%) with high variation at the village level (8.8% - 95.6%) [4]. Seasonal variation has been shown to be an important characteristic of the changing epidemiology of trypanosomiasis on the Jos Plateau, dependent on climatic, husbandry and management factors. The traditional triad of host/vector/parasite factors should be extended to include livestock management and ecological factors such as vegetation, climate and land use to understand the epidemiology of AAT in this area. There is a growing body of evidence supporting the influence of biotic and abiotic factors in the epidemiology of trypanosomiasis in other ecosystems [21,22,35,39,41] and abiotic factors affecting AAT and seasonal variation here highlight a growing trend in pastoral cattle production on the Jos Plateau. The extensive pastoral production system is associated with increased levels of trypanosomiasis in cattle [28,35], in one case twice the prevalence of AAT observed in intensively managed cattle [20]. Malnutrition due to overgrazing by pastoralists was already a problem on the Jos Plateau by the 1970’s [42-44] and since then agricultural expansion and marginalisation of pastoralists has created land use patterns that are a serious threat to the pastoral production system and pastoralist livelihoods. Fulani pastoralists on the Plateau are resorting to increasingly risky measures to maintain their current low-input management practices, malnutrition is widespread and cattle spend ~8 months on migration away from their home village, annually, increasing exposure and susceptibility to disease, security risks and reducing herd productivity and herd management efficiency since cattle must be managed from a distance. The social problems of conflict with farmers persist and increase as pastoralists move into new areas in search of grazing; the current production system is becoming unviable and efforts to intensify production are required. The introduction of dry season feed supplements to reduce dependency for grazing and establishment of bore holes/wells for cattle exclusive use would contribute towards reducing risk of trypanosomiasis and ease friction between farming and pastoral communities.

## Competing interests

The authors declare they have no competing interests and the sponsors had no role in the study design, data collection and analysis, decision to publish, or preparation of the manuscript.

## Authors’ contributions

AM conceived of the study, and participated in its design, coordinated fieldwork, carried out the molecular diagnostic work, analysed data and drafted the manuscript. AF and CD carried out sample collection in the field. KP coordinated laboratory studies. EM, MT, AS and SW participated in study design and coordination, data analysis and helped to draft the manuscript. All authors read and approved the final manuscript.
